# Genetic risk association of CDKN1A and RET gene SNPs with medullary thyroid carcinoma: Results from the largest MTC cohort and meta‐analysis

**DOI:** 10.1002/cam4.2443

**Published:** 2019-08-13

**Authors:** Vasudha Mishra, Pradnya Kowtal, Pallavi Rane, Rajiv Sarin

**Affiliations:** ^1^ Sarin Lab Advanced Centre for Treatment Research and Education in Cancer, Tata Memorial Centre Navi Mumbai India; ^2^ Homi Bhabha National Institute Mumbai India; ^3^ Clinical Research Centre (CRC) Advanced Centre for Treatment Research and Education in Cancer, Tata Memorial Centre Navi Mumbai India; ^4^ Cancer Genetics Clinic, Tata Memorial Hospital Tata Memorial Centre Mumbai India

**Keywords:** CDKN1A, meta‐analysis, MTC, RET, risk association, SNP

## Abstract

**Background:**

Medullary thyroid carcinoma (MTC) is a rare subtype of thyroid cancer. Other than gain‐of‐function *RET* mutations, no other genetic, lifestyle or environmental risk associations have been established for MTC. Several case‐control studies and meta‐analysis have examined the risk association of different SNPs with MTC in different populations but with contradictory or inconclusive results.

**Methods:**

In a large cohort of 438 Indian MTC cases and 489 gender and ethnicity matched healthy controls from 1000 genome project, a comprehensive risk association of 13 SNPs of three pathways—detoxification, cell cycle regulation and *RET* was performed along with meta‐analysis of RET SNPs.

**Results:**

Multivariate logistic regression analysis identified a protective risk association of *CDKN1A*Ser31Arg SNP with both hereditary (OR 0.26; 95% confidence interval [CI] 0.13‐0.55; *P* < .001) and sporadic MTC (OR 0.53; 95% CI 0.36‐0.78; *P* = .001). An increased risk association was identified for *NAT2*Y94Y SNP (OR 1.62, 95% CI 1.17‐2.25, *P* = .004) and *CDKN2A*3′UTR SNP (OR 1.89, 95% CI 1.19‐2.98, *P* = .006*)* with sporadic MTC and *RET* S904S with hereditary MTC (OR 2.82, 95% CI 1.64‐4.86, *P* < .001). Meta‐analysis of *RET* SNPs including our cohort identified increased risk association of all four RET SNPs with MTC.

**Conclusion:**

In this largest SNP risk association study for MTC and the only risk association study of the 13 most commonly studied MTC associated SNPs in a single cohort of this rare cancer, a significant protective risk association of *CDKN1A*Ser31Arg SNP with MTC was shown for the first time. Meta‐analysis identified significant risk association of all four *RET* SNPs, not observed in previous meta‐analysis.

## Graphical abstract

In this largest SNP risk association study for medullary thyroid carcinoma (MTC), a significant protective risk association of *CDKN1A* Ser31Arg SNP with MTC was shown for the first time. Expanded meta‐analysis including our cohort identified significant risk association with four RET SNPs, not observed in previous meta‐analysis.

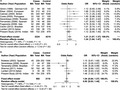

## 1 INTRODUCTION

Thyroid cancers are broadly divided into less aggressive differentiated cancers—Papillary and Follicular thyroid cancer; and very aggressive poorly differentiated cancers—Medullary and Anaplastic Thyroid Cancer. Unlike the more common differentiated Thyroid Cancers, the risk factors for the less common but more aggressive thyroid cancers (medullary thyroid carcinoma [MTC] and ATC) are not well known. MTC originates from the parafollicular C cells of the thyroid. MTC is curable only if it is diagnosed and treated surgically when the disease is confined to the thyroid with or without limited regional nodal spread.^1^ Current systemic treatment including Receptor Tyrosine Kinase Inhibitors such as sorafenib or cytotoxic chemotherapy does not produce long lasting disease control or cure. In the US SEER database, of the 793 MTC cases diagnosed between 1993 and 2002, the 10 year Disease specific survival was 96% for patients with MTC localized to the thyroid, 71% for patients with regional nodal spread and 26% in patients with distant spread.^2^, ^3^, ^4^

Around 75% MTC cases are sporadic while the remaining 25% cases are hereditary in nature and occur as part of an autosomal dominant inherited cancer syndrome called multiple endocrine neoplasia type 2 (MEN2).^5^, ^6^ MEN2 syndrome which affects multiple neuro‐endocrine organs, has three clinical subtypes: MEN2A, MEN2B and Familial MTC.^7^ MTC is the common clinical feature of all the three subtypes.

Mutations in *RET* gene have been identified as the primary susceptibility factor for MTC development. *RET* is a proto‐oncogene that encodes a receptor tyrosine kinase expressed in neural crest derived cells.^8^ In hereditary MTC cases germline point mutations in *RET* are identified in 95%‐98% cases^5^, ^9^, ^10^, ^11^ whereas 40%‐60% sporadic MTC cases have somatic *RET* mutations.^8^, ^12^, ^13^ Other than the high penetrance gain‐of‐function germline or somatic *RET* mutations, no other genetic, lifestyle or environmental risk associations have been clearly established for MTC.

A few small studies which have examined certain lifestyle related risk associations with MTC have either failed to show any risk association or have paradoxically identified a protective role of tobacco smoking and alcohol.^14^, ^15^, ^16^ Several case‐control studies have examined the risk association of SNPs in *RET* and a few other genes involved in xenobiotic metabolism and cell cycle regulation with MTC in different populations.^6^, ^17^, ^18^, ^19^, ^20^, ^21^, ^22^, ^23^, ^24^, ^25^, ^26^, ^27^, ^28^, ^29^, ^30^, ^31^, ^32^, ^33^, ^34^, ^35^, ^36^, ^37^, ^38^ However, most of these studies and their meta‐analysis were either inconclusive or showed contradictory results. The possible reasons for not finding significant and consistent risk association could be the small cohort size of this rare cancer, geo‐ethnic differences or poorly matched controls. Moreover, none of the studies have examined the risk association of SNPs in all these three pathways together in a single cohort. Hence, using the largest cohort of 438 MTC cases (361 sporadic and 77 hereditary) and gender and ethnicity matched 489 healthy controls from the 1000 Genome Project,^39^ South Asian population, a comprehensive analysis of risk association of SNPs in all the three known MTC genetic modifier pathways was undertaken. These include a total of 13 SNPs from genes of detoxification (Cyp1A1m1, Cyp1A2*F, NAT2, GSTP1), cell cycle regulation (*CDKN1A, CDKN1B, CDKN2A, CDKN2B, CDKN2C*) and the *RET* gene (G691S, L769L, S836S, S904S) (Table S1). Further, a meta‐analysis of all the case‐control studies examining risk association of the four *RET* gene SNPs with MTC, including the present study, was conducted to derive definitive conclusions.

## 2 MATERIALS AND METHODS

### 2.1 Study subjects

The study was conducted on 438 Indian MTC cases enrolled between 2006 and 2018 at the Cancer Genetics Clinic; Tata Memorial Hospital as part of Institutional Ethics Committee approved study. Personal and family history with clinico‐pathological details was recorded. Blood sample was collected with written informed consent. The inclusion criteria were histologically confirmed diagnosis of MTC with raised serum calcitonin in patients of any age or gender. Exclusion criteria included a previous history of another cancer except pheochromocytoma which is a part of MEN2 syndrome. The hereditary MTC group consisted of those patients with germline RET proto‐oncogene mutation, irrespective of family history or syndromic features. Those without a germline RET mutation were considered as sporadic MTC. In our cohort of 438 MTC cases, we have 77 hereditary and 361 sporadic MTC cases. Detailed lifestyle or exposure data were not systematically collected and analyzed as their risk association with MTC has not been established in earlier studies. A majority of the large studies on MTC risk association have not taken in to account the demographic or lifestyle factors of MTC patients.^27^, ^28^, ^40^ Genotyping data for healthy controls were extracted from the South Asian population of the 1000 Genome Project (http://www.ensembl.org/Homo_sapiens/Info/Index). This South Asian cohort included all major ethnicities of Indian origin—Punjabis from Lahore, Gujarati from Houston, Telugu from UK, Bengali from Bangladesh and Sri Lankan Tamil from UK.

### 2.2 Molecular genetic testing

#### 2.2.1 *RET* gene sequencing

From the peripheral blood sample, DNA was extracted using Qiagen QIAmp DNA Mini kit (Cat#51304). Germline *RET* mutation analysis was performed for six hotspot exons of *RET* (10, 11, 13, 14, 15 16) using polymerase chain reaction (PCR) and Sanger Sequencing. For PCR, 5 µL (20 ng/µL) gDNA was amplified in a 25 µL PCR reaction volume containing 0.5 µL of each Forward and Reverse primer (10 pmol), 1 µL dNTPs (2.5 mmol), 0.5 µL Taq Polymerase (2 U/µL—Thermo Scientific), 2.5 µL Taq Buffer (10X) and the total volume was adjusted to 25 µL with molecular biology grade water. Primers for PCR were designed using Oligo Explorer version 1.5. Purification of PCR products was done using ExoSAP IT (USB Products, Affimetrix). Sanger Sequencing was performed using BigDye Terminator Cycle Sequencing kit v3.1 (Applied Biosystems) on ABI 3500 and 3730 DNA Sequencer (Applied Biosystems) and electropherograms were analyzed using Chromas Lite version 2.6.4 using reference sequence of *RET* gene extracted from National Center for Biotechnology Information NG_007489.1.

#### 2.2.2 SNP genotyping

SNP genotyping was done using Restriction Fragment Length Polymorphism (RFLP) for 10/13 SNPs. For the remaining three SNPs, genotyping was done using TaqMan as no restriction site for a single cutter restriction enzyme was identified either for the wild type or variant allele. For both genotyping methods, 10% of the genotyping results were confirmed to be true using Sanger Sequencing. SNP genotyping using RFLP was done for Cyp1A1m1, Cyp1A2*F, GSTP1, NAT2, CDKN1A, CDKN1B, CDKN2A, RET L769L, S836S and S904S polymorphisms and using TaqMan for *CDKN2B, CDKN2C* and *RET* G691S polymorphisms. For RFLP, 100 ng gDNA was PCR amplified followed by restriction digestion using reaction conditions as per the manufacturer's protocol. The digested products were visualized on 2% agarose gel and the genotypes were inferred from band sizes in the gel. For TaqMan SNP genotyping, 1 µL gDNA (10 ng/µL) was mixed with 2.5 µL TaqMan universal master mix II with UNG (Applied Biosystems, cat#4440038) and 0.1 µL probe mix (Applied Biosystems) designed for each SNP. TaqMan realtime PCR was performed on QuantStudio 5.0 and genotypes were inferred from amplification plot and allelic discrimination plots. About 5% of all the genotyping results were validated using Sanger Sequencing.

### 2.3 Statistical analysis

All Statistical analysis was performed on SPSS v21.0. SNP genotypes were tested for Hardy‐Weinberg equilibrium (HWE) using Chi‐square HWE test calculator for biallelic markers (http://www.oege.org/software/hwe-mr-calc.shtml) (Table S2). Genotypic frequency was calculated for all 13 SNPs and compared between cases and controls using chi‐square test (Table S3). As the homozygous status of several SNPs was either absent or very low in either cases or controls, analysis was performed only for the dominant model which compares the variant allele either as heterozygous or homozygous form (Aa+aa) with the homozygous wild type allele (AA). Logistic regressions were used to analyze the association between these polymorphisms and MTC risk and odds ratio (ORs) was calculated with 95% confidence interval (CI). All SNPs showing a trend for association on univariate analysis with *P* < .1 were included in the multivariate logistic regression analysis. As multiple comparisons were made for 13 SNPs in a single cohort, a *P*‐value of <.01 was used to consider an association as statistically significant.

### 2.4 Literature search and meta‐analysis

PUBMED search was conducted to identify eligible studies for meta‐analysis using the following search words: “Polymorphism AND MTC”, “SNPs AND MTC”, “*RET* Polymorphisms AND MTC”. All published case‐control studies examining the risk association of these SNPs with sporadic or hereditary MTC were included in the meta‐analysis, the details of which are provided in Table S4. Meta‐Analysis was performed with R‐Software package using minor allele frequency data as the genotype frequencies were not available for several studies. We applied both the fixed effect^41^ and the random effect^42^ model for meta‐analysis. The significance of overall OR was calculated using *Z* test. Heterogeneity between studies was investigated using *I* ^2^ and τ^2^ statistics. The results of meta‐analysis were reported as conventional Forest plots.

## 3 RESULTS

The 438 MTC cases in our cohort included 239 males (54.5%) and 199 (45.4%) females. The mean age at MTC diagnosis was 40.64 ± 14.24, Median: 40 years with the range of 8‐80 years. The 489 controls used for the risk association study included 260 males (53.2%) and 229 females (46.8%). Both the cases and controls were matched for gender (*P* = .67) and ethnicity. The genotype frequencies of all the SNPs included in the study are summarized in Table S2. HWE was maintained for all 13 SNPs in the controls and for 11/13 SNPs in the MTC cases (Table S2).

### 3.1 Risk associations

#### 3.1.1 Present study

On univariate logistic regression analysis, *CDKN1A* SNP showed consistent and significant association with reduced risk of MTC in both hereditary (OR = 0.52; 95% CI = 0.27‐0.99; *P* = .048) and sporadic MTC groups (OR = 0.63; 95% CI = 0.45‐0.88; *P* = .007). The variant allele A was overrepresented in the control population (26.2%) as compared to both the hereditary cases (15.6%) and the sporadic cases (18.3%) (Tables 1 and 3). The strong association of CDKN1A SNP with reduced MTC risk was further confirmed on multivariate logistic regression analysis in both the hereditary (OR = 0.27; 95% CI = 0.13‐0.55; *P* < .001) and sporadic MTC groups (OR = 0.53; 95% CI = 0.36‐0.78; *P* = .001) (Table 2 and 4).

**Table 1:** Univariate logistic regression analysis for association between SNPs and risk of hereditary MTC development (hMTC: hereditary MTC; Wt: Wild type; Hz: Heterozygous; Hm: Homozygous)

Gene/SNP	Genotype frequency—hMTC cases (n = 77)		Genotype frequency—Controls (n = 489)		OR	95% CI	*P*‐value
	Wt	Hz + Hm	Wt	Hz + Hm			
Cyp1A1m1	37 (48.1%)	40 (51.9%)	218 (44.6%)	271 (55.4%)	0.870	0.537‐1.407	.570
Cyp1A2	28 (36.4%)	49 (63.6%)	145 (29.7%)	344 (70.3%)	0.738	0.446‐1.220	.236
NAT2	31 (42.2%)	46 (59.7%)	167 (34.2%)	322 (65.8)	0.770	0.470‐1.259	.297
GSTP1	35 (45.5%)	42 (54.5%)	251 (51.3%)	238 (48.7%	1.266	0.781‐2.050	.339
**CDKN1A**	**65 (84.4%)**	**12 (15.6%)**	**361 (73.8%)**	**128 (26.2%)**	**0.521**	**0.272‐0.995**	**.048**
CDKN1B	35 (45.5%)	42 (54.5%)	224 (45.8%)	265 (54.2%)	1.014	0.626‐1.643	.954
CDKN2A	67 (87%)	10 (13%)	439 (89.7%)	50 (10.2%)	1.310	0.634‐2.708	.465
CDKN2B	46 (59.8%)	31 (40.2%)	266 (54.4%)	223 (45.6%)	0.804	0.493‐1.311	.382
CDKN2C	62 (80.5%)	15 (19.5%)	387 (79.1%)	102 (20.8%)	0.918	0.501‐1.680	.781
G691S RET	37 (48.1%)	40 (51.9%)	283 (57.8%)	206 (42.1%)	1.485	0.917‐2.404	.108
L769L RET	35 (45.5%)	42 (54.5%)	178 (36.4%)	311 (36.6%)	0.687	0.423‐1.115	.129
S836S RET	64 (83.1%)	13 (16.9%)	411 (84%)	78 (15.9%)	1.070	0.562‐2.037	.836
S904S RET	36 (46.8%)	41 (53.2)	285 (58.3%)	204 (41.7%)	1.591	0.982‐2.578	.06

Values in bold indicates significant associations.

Abbreviations: CI, confidence interval; MTC, medullary thyroid carcinoma; OR, odds ratio.

**Table 2:** Multivariate logistic regression analysis for association between SNPs and risk of hereditary MTC (hMTC) development (SNPs with significance <0.1 from univariate analysis were included in multivariate analysis)

Gene/SNP	Genotype frequency—hMTC cases (n = 77)		Genotype frequency—Controls (n = 489)		OR	95% CI	*P*‐value
	Wt	Hz + Hm	Wt	Hz + Hm			
CDKN1A	65 (84.4%)	12 (15.6%)	361 (73.8%)	128 (26.2%)	0.266	0.129‐0.549	***<.001***
S904S RET	36 (46.8%)	41 (53.2)	285 (58.3%)	204 (41.7%)	2.821	1.636‐4.862	***<.001***

*P*‐value in bold indicates significant associations.

Abbreviations: CI, confidence interval; MTC, medullary thyroid carcinoma; OR, odds ratio.

Multivariate logistic regression analysis also identified significant risk association for the *RET* S904S SNP in the hereditary MTC group (OR = 2.82; 1.64‐4.86; *P* < .001) (Table 2) whereas for *CDKN2A* (OR = 1.89; 95% CI = 1.20‐2.98; *P* = .006) and *NAT2* SNP (OR = 1.62; 95% CI = 1.17‐2.25; *P* = .004) in the sporadic MTC group (Table 2).

#### 3.1.2 Meta‐analysis including present study

**Figure 1:**
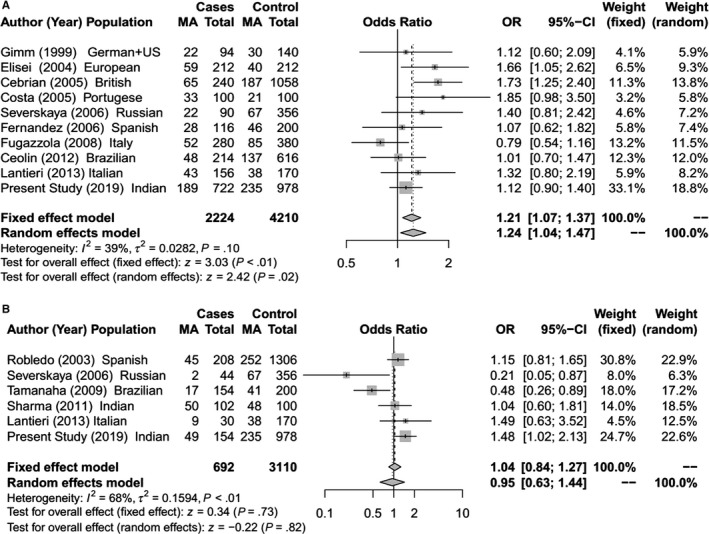
Forest Plot for meta‐analysis on allelic association of *RET* G691S SNP with (A) Sporadic MTC; (B) Hereditary MTC [The total for cases and controls are allelic count (2n)]

**Figure 2:**
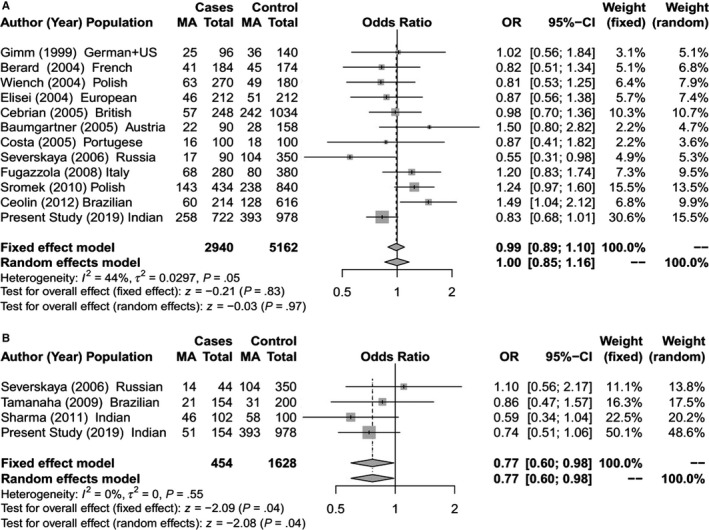
Forest Plot for meta‐analysis on allelic association of *RET* L769L SNP with (A) Sporadic MTC; (B) Hereditary MTC [The total for cases and controls are allelic count (2n)]

**Figure 3:**
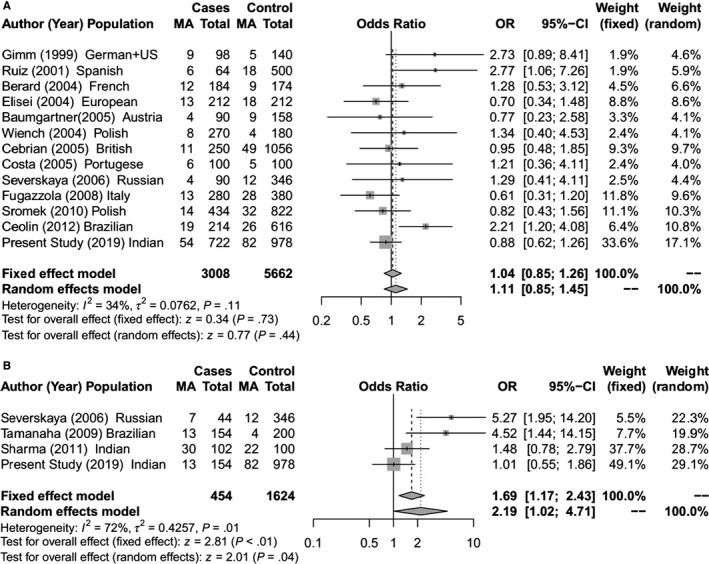
Forest Plot for meta‐analysis on allelic association of *RET* S836S SNP with (A) Sporadic MTC; (B) Hereditary MTC [The total for cases and controls are allelic count (2n)]

**Figure 4:**
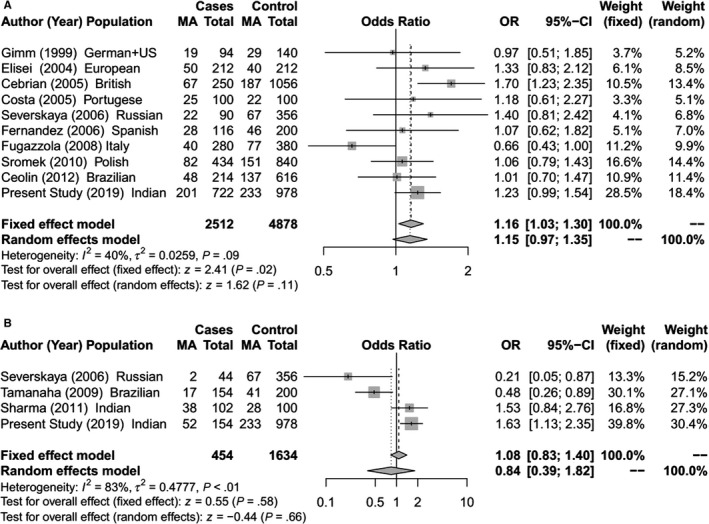
Forest Plot for meta‐analysis on allelic association of *RET* S904S SNP with (A) Sporadic MTC; (B) Hereditary MTC [The total for cases and controls are allelic count (2n)]

We identified 23 case‐control studies examining risk associations of one or more of these 13 SNPs with MTC. However, for nine SNPs in the cell cycle regulation (*CDKN1A, CDKN1B, CDKN2A, CDKN2B, CDKN2C*) and detoxification pathway (CYP1A1m1, CYP1A2*F, NAT2, GSTP1), only single small cohort studies had examined their risk association with MTC.^23^, ^25^, ^26^, ^27^ Hence the meta‐analysis was performed only for the four *RET* gene SNPs (G691S, L769L, S836S, S904S) one or more of which are reported in 19 case‐control studies. This included a total of 346 cases and 1555 controls in the hereditary MTC group and 1640 cases and 2968 controls in sporadic MTC group (Table S4). The ORs with 95% CIs calculated for the allelic distribution of SNPs for each study is shown in their respective Forest plots (Figures 1, 2, 3, 4).

The meta‐analysis identified a significant association between *RET* L769L and S836S SNPs with risk of hereditary MTC (Figures 2B and 3B).The *RET* S836S variant allele was found to be associated with increased susceptibility to MTC. The effect was observed under both the fixed effect model (OR = 1.69; 95% CI = 1.17‐2.43; *P* < .01) and random effect model (OR = 2.19; 95% CI = 1.02‐4.71; *P* = .04).For *RET* L769L variant, a significant protective risk association with MTC was observed under both fixed effect model (OR = 0.77; 95% CI = 0.60‐0.98; *P* = .04) and random effect model (OR = 0.77; 95% CI = 0.60‐0.98; *P* = .04). Further in the sporadic MTC group, meta‐analysis showed significantly increased risk of MTC with the *RET* G691S and S904S (Figures 1A and 4A). For G691S, the association was observed under both fixed effect model (OR = 1.21 95% CI = 1.07‐1.37; *P* < .01) and random effect model (OR = 1.24; 95% CI = 1.04‐1.47; *P* = .02). For S904S, this effect was observed under only fixed effect model (OR = 1.16; 95% CI = 1.03‐1.30; *P* = .02).

## 4 DISCUSSION

In hereditary cancer syndromes, highly penetrant germline mutations in proto‐oncogene or tumor suppressor genes confer a very high lifetime risk of cancer development.^17^ However in several sporadic cancers, in addition to environmental or lifestyle factors there is a component of weak genetic susceptibility conferred by low penetrance genetic variants. While there are no clearly established lifestyle or environmental risk factors for susceptibility to MTC, several SNPs in *RET* as well as other genes have been reported to slightly increase or decrease the risk of MTC development.^18^, ^26^, ^30^, ^36^, ^43^ However, the findings of these studies are inconsistent. Of the four previously reported meta‐analysis of *RET* gene SNPs,^6^, ^17^, ^28^, ^32^ two demonstrated a significant risk association of *RET*G691S SNPs with MTC.^17^, ^28^ No other significant risk association has been observed in the other two studies.

Like previous case‐control studies in MTC,^43^ we have analyzed the risk association of SNPs independently in the hereditary and sporadic MTC groups for our cohort as well as for the meta‐analysis. In our cohort, multivariate logistic regression analysis identified a highly significant (*P* < .01) protective risk association of *CDKN1A* SNP for hereditary MTC as well as sporadic MTC (Tables 1, 2, 3, 4). Two SNPs (*NAT2* and *CDKN2A)* had a significant increased risk association with sporadic MTC (Table 2) while another SNP (*RET* S904S) had a significant increased risk association with hereditary MTC (Table 2). With the inclusion of 346 hereditary MTC cases in the meta‐analysis for 4 *RET* gene SNPs, a significant protective risk association was observed for *RET* L769L SNP while a significant increased risk association was seen with *RET* S836S SNP (Figure 2B and 3B). For the 1640 sporadic cases included in the meta‐analysis, significant increased risk association was seen for the *RET* G691S and S904S SNPs (Figures 1A and 4A). A few functional and in‐silico studies have postulated and examined how different *RET* SNPs modulate the risk of MTC development. These include their effect on RNA stability or its expression, creation of a new alternative splicing site^18^, ^21^, ^22^, ^36^ or changes in phosphorylation sites.^17^ However, the findings of these studies have been inconclusive.

**Table 3:** Univariate logistic regression analysis for association between SNPs and risk of sporadic MTC development (sMTC: sporadic MTC)

Gene/SNP	Genotype frequency—sMTC cases (n = 361)		Genotype frequency—Controls (n = 489)		OR	95% CI	*P*‐value
	Wt	Hz + Hm	Wt	Hz + Hm			
Cyp1A1m1	161 (44.6%)	200 (55.4%)	218 (44.6%)	271 (55.4%)	0.999	0.760‐1.314	.996
Cyp1A2	117 (32.4%)	244 (67.6%)	145 (29.7%)	344 (70.3%)	0.879	0.655‐1.179	.390
NAT2	103 (28.5%)	258 (71.5%)	167 (34.2%)	322 (65.8)	1.299	0.967‐1.745	.082
GSTP1	208 (57.6%)	153 (42.4%)	251 (51.3%)	238 (48.7%	0.776	0.590‐1.020	.069
**CDKN1A**	**295 (81.7%)**	**66 (18.3%)**	**361 (73.8%)**	**128 (26.2%)**	**0.631**	**0.452‐0.882**	**.007**
CDKN1B	182 (50.4%)	179 (49.6%)	224 (45.8%)	265 (54.2%)	0.831	0.633‐1.092	.184
CDKN2A	310 (85.9%)	51 (14.1%)	439 (89.7%)	50 (10.2%)	1.444	0.953‐2.190	.083
CDKN2B	206 (57%)	155 (42.9%)	266 (54.4%)	223 (45.6%)	0.898	0.682‐1.180	.439
CDKN2C	297 (82.2%)	64 (17.7%)	387 (79.1%)	102 (20.8%)	0.818	0.578‐1.157	.256
G691S RET	201 (55.7%)	160 (44.3%)	283 (57.8%)	206 (42.1%)	1.094	0.831‐1.439	.523
L769L RET	146 (40.4%)	215 (59.6%)	178 (36.4%)	311 (36.6%)	0.843	0.637‐1.115	.231
S836S RET	311 (86%)	50 (13.9%)	411 (84%)	78 (15.9%)	0.847	0.577‐1.244	.398
S904S RET	194 (53.7%)	167 (46.3%)	285 (58.3%)	204 (41.7%)	1.203	0.914‐1.582	.187

Values in bold indicates significant associations.

Abbreviations: CI, confidence interval; MTC, medullary thyroid carcinoma; OR, odds ratio.

**Table 4:** Multivariate logistic regression analysis for association between SNPs and risk of sporadic MTC (sMTC) development (SNPs with significance <0.1 from univariate analysis were included in multivariate analysis)

Gene/SNP	Genotype frequency—sMTC cases (n = 361)		Genotype frequency—Controls (n = 489)		OR	95% CI	*P*‐value
	Wt	Hz + Hm	Wt	Hz + Hm			
NAT2	103 (28.5%)	258 (71.5%)	167 (34.2%)	322 (65.8)	1.622	1.168‐2.251	**.004**
GSTP1	208 (57.6%)	153 (42.4%)	251 (51.3%)	238 (48.7%	0.741	0.540‐1.018	.065
CDKN1A	295 (81.7%)	66 (18.3%)	361 (73.8%)	128 (26.2%)	0.526	0.357‐0.776	**.001**
CDKN2A	310 (85.9%)	51 (14.1%)	439 (89.7%)	50 (10.2%)	1.888	1.197‐2.978	**.006**

Values in bold indicates significant associations.

Abbreviations: CI, confidence interval; MTC, medullary thyroid carcinoma; OR, odds ratio.

Univariate and multivariate logistic regression analysis in our cohort also demonstrated a strong protective association between *CDKN1A* SNP with hereditary and sporadic MTC. The *CDKN1A* gene, also known as p21^CIP1/WAF1^, encodes a cyclin‐dependent kinase inhibitor which binds to and inhibits the activity of Cyclin‐CDK2 or CDK4 complexes regulating cell cycle progression at G1 stage.^44^, ^45^ *CDKN1A* activity is regulated by p53 which binds to its promoter and induces cell cycle arrest in response to various stimuli.^45^ This gene is often deregulated in human cancers with altered expression reported in several cancers including cervical, breast, ovarian, liver, uterine, and head and neck cancers.^46^ The *CDKN1A* SNP (rs1801270) at codon 31 (Ser31Arg) reported in the present study falls in a highly conserved N‐terminal region of the protein, which is demonstrated to contain tumor suppressor function.^44^ Functional studies suggested that while the *CDKN1A*‐Ser and Arg variant possess similar kinase inhibitory and growth suppression abilities,^47^ their transcriptional efficiency is significantly different.^48^ The allelic frequency of this SNP varies significantly among different populations with minor allele frequency of 15% in the South Asian Population (1000 Genome Project). Several molecular epidemiological studies of *CDKN1A* Ser31Arg SNP show conflicting results with some studies reporting increased risk association with tobacco related upper aerodigestive tract cancers,^49^ while showing a protective effect in human papilloma virus related cervical cancers.^50^, ^51^ The only study of this SNP in MTC has been reported by Barbieri et al^27^ in a small cohort of 45 sporadic MTC cases. Even though no significant risk association for MTC development was identified, perhaps due to the small sample size, extrathyroidal tumor extension was significantly less in patients with the *CDKN1A* SNP as compared to those with wild type *CDKN1A* (50% versus 92%, *P* = .037). In our study of much larger cohort of this rare cancer, univariate and multivariate logistic regression analysis shows the highly significant protective effect of *CDKN1A* SNP on risk of MTC development in sporadic as well as hereditary MTC.

The significant risk association of the variant allele C of *CDKN2A* 3’UTR SNP (rs11515), identified in our sporadic MTC cohort has also been reported as a risk allele in a Brazilian cohort of 45 sporadic MTC by Barbieri et al in 2014.^27^ We have also identified a significantly increased risk association of the variant allele T of the *NAT2* Y94Y SNP (rs1041983) in our sporadic MTC cohort, as reported previously in a Brazilian cohort of 132 hereditary MTC cases.^26^ However the same Brazilian group in their cohort of 47 sporadic MTC cases, found the variant allele T of this NAT2 SNP to be protective. This could be due to the small cohort size or difference in the frequency of alleles in the admixture population.^25^

The significant risk association of *CDKN2A*3’UTR SNP (rs11515) identified in our sporadic MTC cohort has also been reported in a Brazilian cohort of 45 sporadic MTC by Barberi et al in 2014.^27^ For the *NAT2* Y94Y SNP (rs1041983) we identified a significantly increased risk association of the variant T allele in 361 sporadic MTC cases, as previously reported in a Brazilian cohort of 132 hereditary MTC cases.^26^ Paradoxically, in a study with 47 sporadic MTC cases, reported from the same Brazilian group,^25^ the wild type C allele was associated with increased risk of MTC, the reasons for which have not been elaborated.

This is the first study to examine the MTC risk association of 13 different SNPs in genes of three distinct pathways in a single cohort, which is also the largest cohort of this rare cancer reported so far. The meta‐analysis conducted by us, with the inclusion of MTC cases from our cohort, has increased the total sporadic MTC cases to 1640 and hereditary MTC cases to 346 (Table S4). While the previous meta‐analysis by Figlioli et al in 2013 had failed to identify significant risk association with any of these four *RET* SNPs,^6^ in our expanded meta‐analysis cohort, we could identify significant risk association of *RET* L769L and S836S in hereditary MTC and of G691S and S904S in sporadic MTC.

One of the limitations of our study is that unlike classical case‐control studies, instead of recruiting and genotyping matched controls, we used healthy gender and ethnicity matched South Asian controls from the 1000 genome database. Matching for age was not possible as MTC, especially the hereditary MTC, is known to occur in childhood and recruiting minor subjects as healthy controls for genotyping study raises ethical issues. Of all the MTC case‐control studies, some have not reported whether controls were matched^22^, ^43^ whereas many have failed to obtain controls matched for age or gender.^24^, ^26^, ^28^ Moreover, in the absence of a clearly established lifestyle or environmental factors for MTC risk, none of the MTC SNP case‐control studies have described or matched for these factors, as is the case in our study.

Taken together, the findings from comprehensive genotyping of 13 SNPs in our large MTC cohort, we showed for the first time, a significant protective risk association of *CDKN1A* SNP (rs1801270) with MTC and through meta‐analysis of expanded cohort, we also showed a risk association of four *RET* SNPs with MTC. Identification of one or more low penetrance alleles in risk association studies in diverse cancers could provide some biological insight into cancer development but are not useful as biomarkers of prognosis or predisposition. However study of a large number of low penetrance alleles in large case‐control studies could help in developing polygenic risk scores. The present study therefore underscores the need for large replicative risk association studies using a control group from the local population with well‐defined characteristics to understand the molecular mechanisms through which these low penetrance alleles modulate MTC risk.

## CONFLICT OF INTEREST

The authors declare no conflict of interest.

## Data Availability

The data that support the findings of this study are available from the corresponding author upon reasonable request.
