# Supplementary material for: Genetic risk association of CDKN1A and RET gene SNPs with medullary thyroid carcinoma: Results from the largest MTC cohort and meta‐analysis
Source: Cancer Med. 2019 Aug 13;8(13):6151–61. doi: 10.1002/cam4.2443 (PMC6792509; doi:10.1002/cam4.2443)
Supplement: Supplementary file 1 [file CAM4-8-6151-s001.docx]

**Supplementary Table S1**: List of SNPs of genes of distinct pathways selected for the study based on detailed literature search


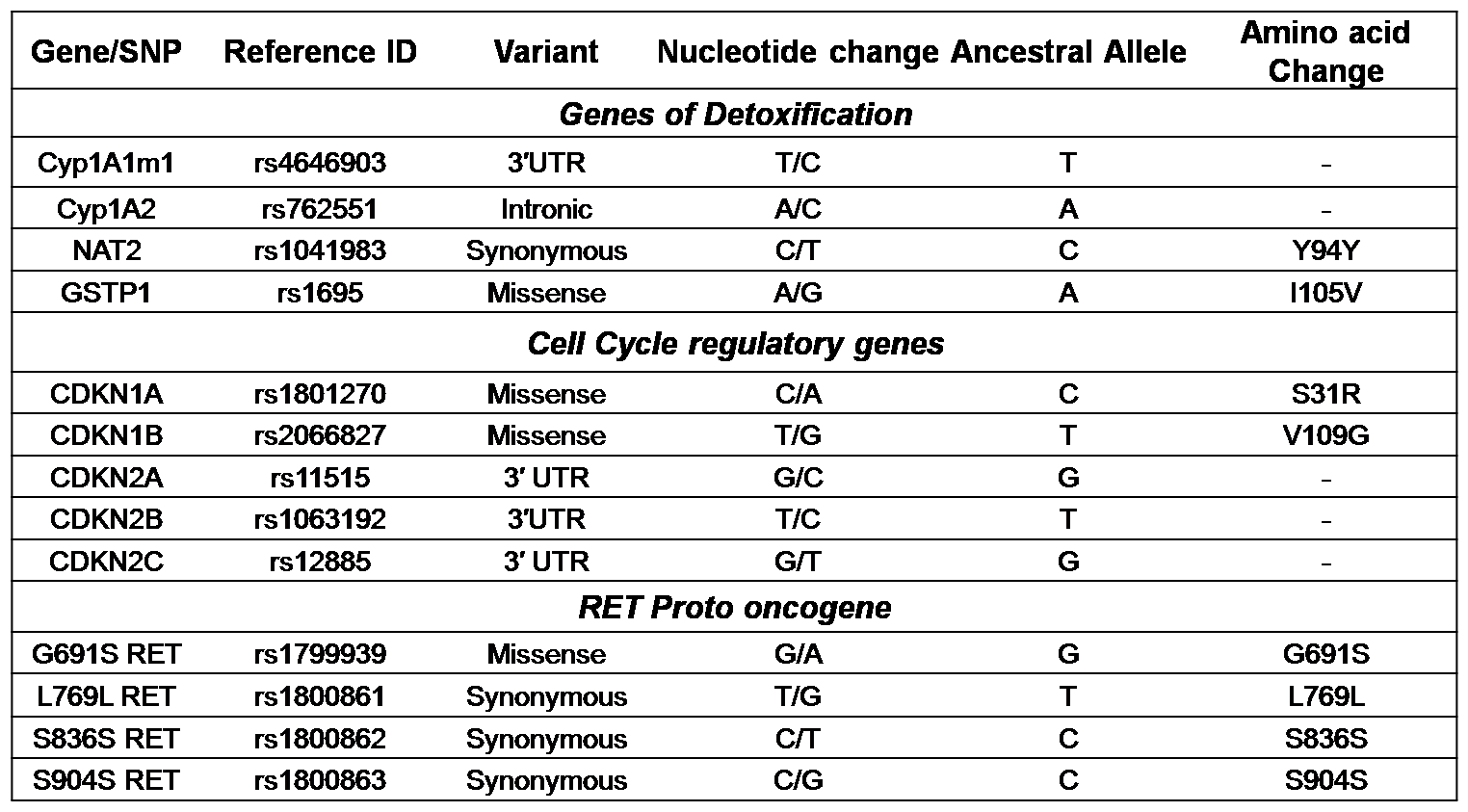


**Supplementary Table S2**: Hardy-Weinberg Equilibrium (HWE) Calculations for Cases (n=438) and Control (n=489) [MAF: Minor Allele Frequency]


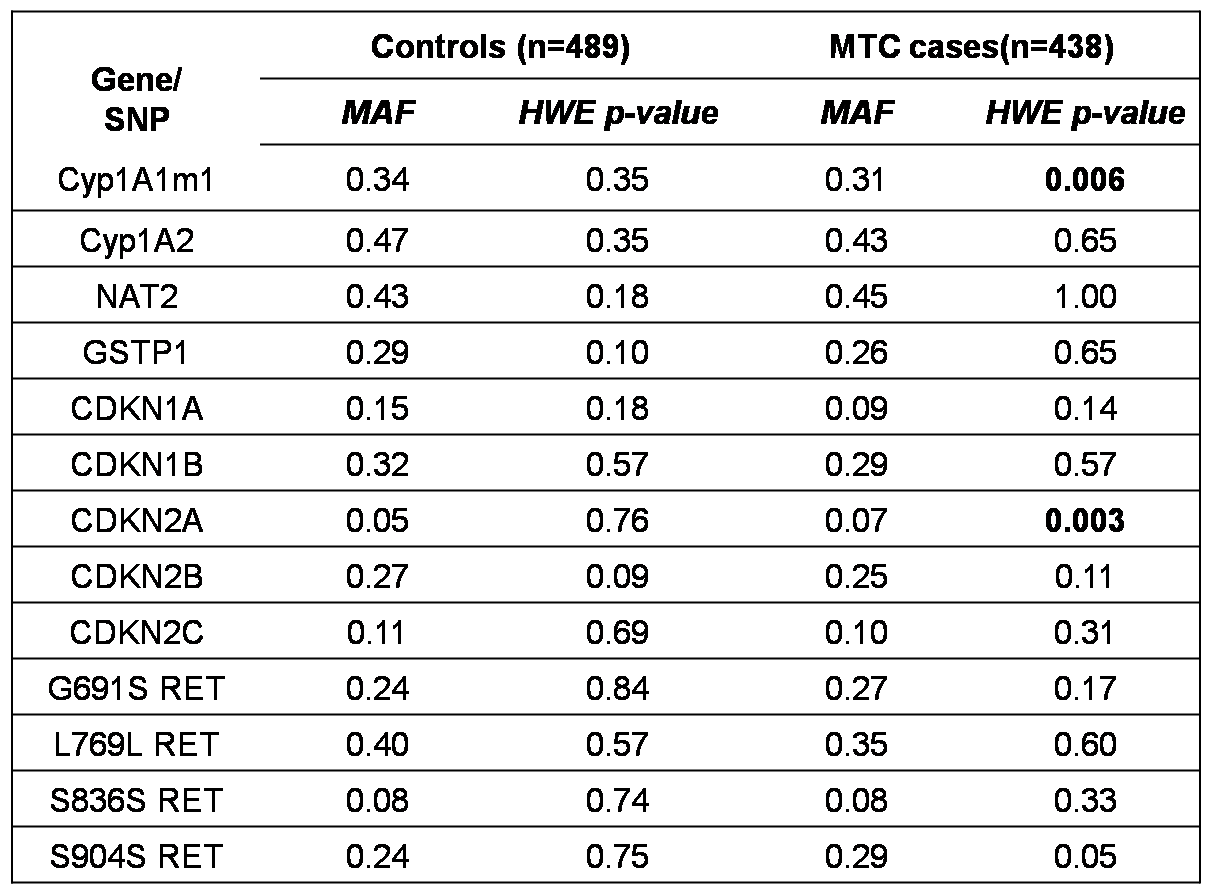


**Supplementary Table S3**: Genotype frequency comparison between: **(A):** hereditary MTC (hMTC) cases and controls; **(B):** sporadic MTC (sMTC) cases and controls

**(A)**


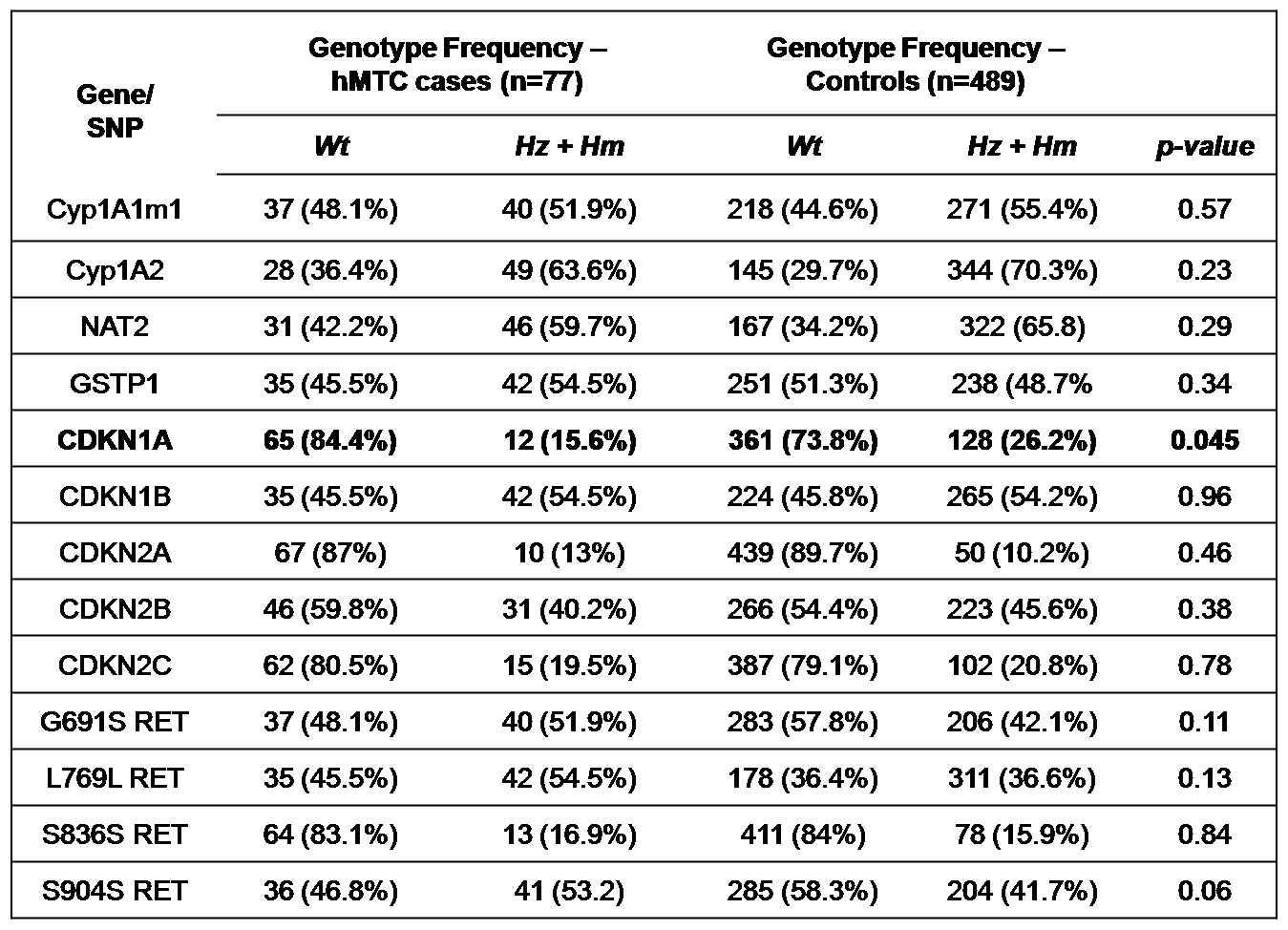


**(B)**
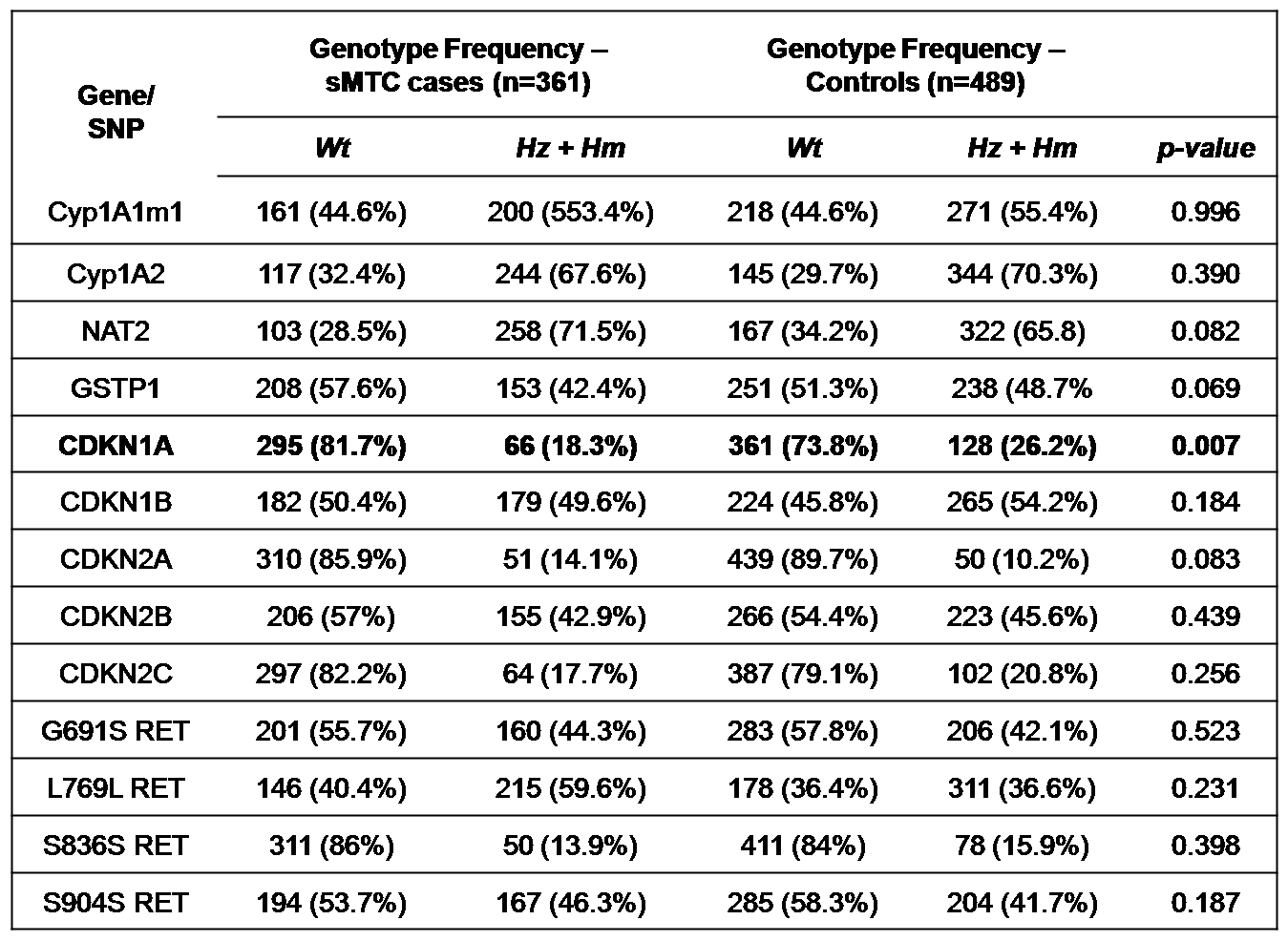


**Supplementary Table S4: (A):** Details on sporadic MTC studies included in the meta-analysis. **(B):** Details on hereditary MTC studies included in the meta-analysis.

**(A)**

**
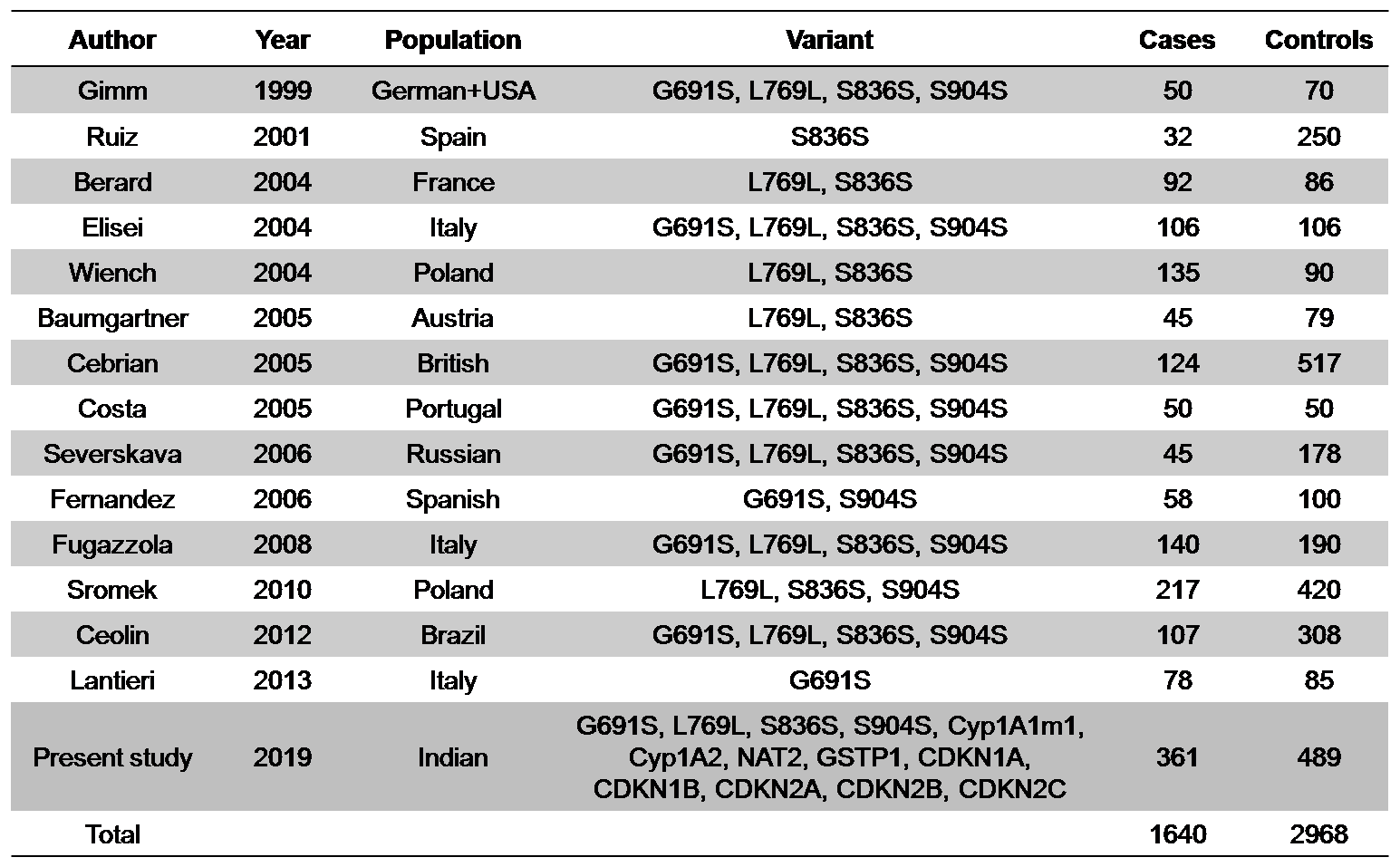
**

**(B)**

**
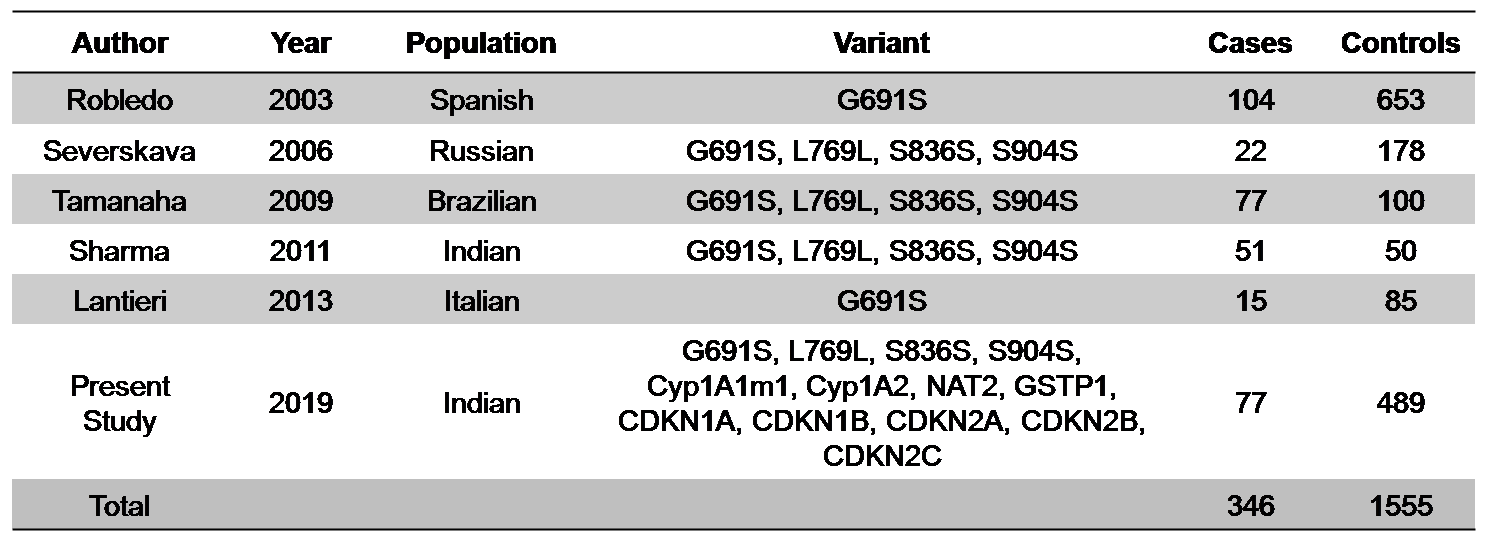
**
